# Nitrogen fertilization rates mediate rhizosphere soil carbon emissions of continuous peanut monoculture by altering cellulose-specific microbess

**DOI:** 10.3389/fpls.2023.1109860

**Published:** 2023-03-03

**Authors:** Zhengfeng Wu, Zhaohui Tang, Tianyi Yu, Jiancheng Zhang, Yongmei Zheng, Jishun Yang, Yue Wu, Qiqi Sun

**Affiliations:** ^1^ Shandong Peanut Research Institute, Shandong Academy of Agricultural Sciences, Qingdao, China; ^2^ Institute of Crop Germplasm Resources, Shandong Academy of Agricultural Sciences, Jinan, China

**Keywords:** microbes, soil carbon, N fertilization, rhizosphere, peanut

## Abstract

**Introduction:**

Crops influence both soil microbial communities and soil organic carbon (SOC) cycling through rhizosphere processes, yet their responses to nitrogen (N) fertilization have not been well investigated under continuous monoculture.

**Methods:**

In this study, rhizosphere soil microbial communities from a 5-year continuous mono-cropped peanut land were examined using Illumina HighSeq sequencing, with an N fertilization gradient that included 0 (N0), 60 (N60), 120 (N120) and 180 (N180) kg hm^−2^. Soil respiration rate (*R*_s_) and its temperature sensitivity (*Q*_10_) were determined, with soil carbon-acquiring enzyme activities assayed.

**Results and discussion:**

The obtained results showed that with N fertilization, soil mineral N (N_min_) was highly increased and the soil C/N ratio was decreased; yields were unchanged, but root biomass was stimulated only at N120. The activities of β-1,4-glucosidase and polyphenol oxidase were reduced across application rates, but that of β-1,4-cellobiohydrolase was increased only at N120. Bacterial alpha diversity was unchanged, but fungal richness and diversity were increased at N60 and N120. For bacterial groups, the relative abundance of Acidobacteria was reduced, while those of Alphaproteobacteria and Gammaproteobacteria were increased at N60 and N120. For fungal members, the pathogenic Sordariomycetes was inhibited, but the saprotrophic Agaricomycetes was promoted, regardless of N fertilization rates. RDA identified different factors driving the variations in bacterial (root biomass) and fungal (N_min_) community composition. N fertilization increased *R*_s_ slightly at N60 and significantly at N120, mainly through the promotion of cellulose-related microbes, and decreased *R*_s_ slightly at N180, likely due to carbon limitation. N fertilization reduced microbial biomass carbon (MBC) at N60, N120 and N180, decreased SOC at N120 and N180, and suppressed dissolved organic carbon (DOC) at N180. In addition, the unchanged *Q*_10_ may be a joint result of several mechanisms that counteracted each other. These results are of critical importance for assessing the sustainability of continuously monocultured ecosystems, especially when confronting global climate change.

## 1 Introduction

Soil CO_2_ emissions in agricultural land contribute up to 10.3% of total greenhouse gas emissions (Lal, 2004). As the main method of agriculture, intensive agriculture is challenged by soil degradation and environmental pollution due to monoculture and excessive N fertilization, which are not conducive to the sustainability of agroecosystems. Continuous monocropping not only decreases crop yield but also results in pathogen accumulation, and this phenomenon is known as the “continuous cropping obstacle” (Chen et al., 2018), yet the subsequent carbon (C) effect has not been well elucidated. Rhizosphere processes are one of the most important ways in which plants affect C cycling in terrestrial ecosystems (Zhu et al., 2014; Zhao et al., 2022). Rhizosphere microbial communities thrive on constant influxes of root exudates (Deng et al., 2015), the relevance of which is important for C turnover (Zhang et al., 2022b). Considering the increasing importance of microbial-mediated SOC decomposition in global SOC cycling (Zhao et al., 2022), there is an urgent need to investigate rhizosphere microbial communities and their contribution to soil C dynamics. Moreover, small changes in rhizosphere microbiome composition predict disease outcomes earlier than pathogen density variations (Gu et al., 2022). Therefore, it is of great agronomic interest to understand the alterations in rhizosphere microbial communities and their functions in continuous monocropping ecosystems.

Rhizosphere microbial communities are highly influenced by N fertilization. On the one hand, long-term N fertilization can enhance crop yield and stability (Liang, 2022). On the other hand, excessive N fertilizer not only cannot stimulate crop yield but also aggravates soil CO_2_ emissions (Zhang et al., 2020) and is currently one of the major issues affecting agricultural production in China (Dong et al., 2021). N fertilization can either aggravate (Zhang et al., 2022a) or alleviate (Fu et al., 2020) continuous cropping obstacles by affecting rhizosphere microbes, inevitably exerting a discernible effect on rhizosphere soil C dynamics. Generally, N fertilization directly influences soil microbial growth, diversity and activities (respiration rate and enzyme activities) by increasing soil mineral N availability (Zeng et al., 2016), especially under N-limited conditions. Indirectly, N fertilization affects microbial community composition by changing soil pH (Dong et al., 2021) and/or by regulating the belowground allocation of photosynthesis (Ning et al., 2021). Furthermore, N fertilization impacts the microbial utilization of photosynthesized C by decreasing soil C/N, which is highly related to substrate quality (Leifeld and von Lutzow, 2014). All these differences can result in altered SOC decomposition and accumulation (Zhou et al., 2022). In addition, N fertilization may have contrasting effects on the rhizosphere and bulk- soil C stocks (Zhu et al., 2020). Neglecting the regulation of rhizosphere C stocks may be biased when estimating the potential for soil C emission, and the rhizosphere processes should be properly accounted for. To date, the mechanism by which rhizosphere soil microbes and associated C dynamics respond to N fertilization from continuous monocropping ecosystems has not been investigated. This matter is of great significance for the sustainable development of such intensive agroecosystems, especially when confronting future climate change.

Legume cultivation is an important option to sequester SOC for sustainable food production and environmental restoration, as legume-based biological N fixation contributes approximately 50−70 terrograms (Tg) N hectare (ha)^−1^ globally and strongly impacts SOC sequestration (Virk et al., 2022). Peanut (*Arachis hypogaea L*.) is an important food legume, and its perpetual production is crucial for edible oil security (Xiong et al., 2013). In recent years, peanut yields and quality have been seriously compromised by continuous cropping obstacles and excessive use of N fertilizer. N fixation by nodules varies with N fertilization rates (Wang et al., 2016), which strengthens the complexity of crop-soil-microbial interactions. This study was conducted in a 5-year continuous peanut monocropping system from 2017 to 2021. Rhizosphere soil samples were collected from peanut plants grown in soil subjected to four levels of N fertilization in the pod setting stage in 2021. Our aims were 1) to investigate the response patterns of rhizosphere soil microbial communities and their functions (soil respiration, *Q*_10_ and carbon-acquiring enzyme activities) to N fertilization, and 2) to elucidate the influencing mechanism of N fertilization on rhizosphere soil C dynamics. We hypothesized that 1) rhizosphere soil microbial communities would respond significantly to N fertilization, given the sensitivity of microbes to environmental disturbance, and 2) *R*_s_ would be accelerated and *Q*_10_ would be reduced, due to N-induced improvement of substrate quantity and quality.

## 2 Materials and methods

### 2.1 Experimental design and soil sampling

An *in situ* N fertilization experiment has been conducted since 2017 at the Laixi experimental station of Shandong Peanut Research Institute, China (36°48′47″N, 120°30′17″E), with maize as the preceding crop and peanut monocropping every year since 2017. The study area is characterized by a semihumid monsoon climate, with a mean annual rainfall of 732 mm and a daily air temperature of 11.3°C. During the peanut growing periods, the mean precipitation, effective accumulated temperature and sunshine duration were 404 mm, 3068°C and 852 h, respectively. The sampled soil is sandy brown soil, with soil basic nutrients in the 0−30 cm layers as follows: soil organic matter 11.9 g kg^−1^, soil N 1.22 g kg^−1^, available N 78.2 mg kg^−1^, available phosphorus (P) 45.3 mg kg^−1^, available potassium (K) 97.6 mg kg^−1^, and pH 5.62. A peanut variety, Huayu 22 (HY22), which is widely planted in major peanut-producing areas of Shandong Province due to its high yields, nice fruit shape and comprehensively strong stress-resistance, was used in the experimental planting. According to peanut production management practices, the recommended N application rate of HY22 was 120 kg hector-meter (hm)^−2^ during the growing season, and the N application treatments selected in this study were 50%, 100% and 150% of the recommended N application rate: 0 kg hm^−2^ (N0), 60 kg hm^−2^ (N60), 120 kg hm^−2^ (N120) and 180 kg hm^−2^ (N180), respectively. Each treatment was replicated three times; hence, 12 experimental plots were completely randomized and sampled during the experimental period. To prevent leakage of dissolved fertilizer, each plot was separated with plexiglass boards, which were inserted 100 cm belowground. The peanuts were planted in ridges and furrows under plastic film mulching, sown in May and harvested in September. Three peanut seeds were sown in each hole, and two plants with similar growth were retained after the seedling stage. Urea (N≥46%) was the only type of N fertilizer. The application rates of P (P_2_O_5_, 90 kg hm^−2^) and K (K_2_O, 120 kg hm^−2^) fertilizers were the same in all experimental plots, and 100% N, P and K fertilizers were applied to the 0−20 cm soil layer before seeding. Rhizosphere soil samples were collected at the podding stage in August 2021, during which peanut plants grow vigorously and biomass accumulates quickly. Each complete peanut plant was removed with its immediate block of soil included. The soil tightly attached to the roots was brushed off gently with a sterile brush and gathered as rhizosphere soil. Three replicate samples of rhizosphere soil were collected from plants (four peanut plants in each plot were randomly selected) subjected to each N fertilization treatment. All samples were brought to the laboratory on ice packs. The soils were sieved through 2-mm meshes to remove fine roots, residues and stones. Each sample was divided into three parts: one part was stored at −80°C for DNA extraction; the second part was stored at 4°C for measurement of soil respiration rate (*R*_s_), soil mineral N (N_min_), microbial biomass C, N (MBC, MBN) and activities of soil carbon-acquiring enzymes; and the third part was air-dried to determine SOC content, soil total nitrogen content (TN) and soil pH.

### 2.2 Soil respiration rate (*R*_s_) and *Q*_10_

Fifty grams of fresh soil was placed into a 500-ml butyl lithium bottle and incubated at six temperature gradients: 5°C, 10°C, 15°C, 20°C, 25°C and 30°C, to systematically measure the CO_2_ emission rate of each treatment and their sensitivity to temperature changes. According to the previously described incubation method (Lefèvre et al., 2014; Ding et al., 2017), the fresh samples were preincubated with lids open at 20°C for 7 days to stabilize microbial activities. Once the formal experiment started, 50 grams of incubated soil was placed into a butyl lithium bottle, accompanied by a plastic bottle containing 20 ml of 0.1 M NaOH solution to absorb the released CO_2_, with three replicates. In addition, three plastic bottles containing 20 ml of 0.1 M NaOH solution with no soil sample were included to calculate the CO_2_ absorbed from air. All bottles were incubated with lids closed, first at 5°C for 48 h, then each at 10°C, 15°C, 20°C and 25°C for 24 h, and finally at 30°C for 12 h. The prolonged incubation period at the lowest temperature ensured adequate time to accumulate detectable changes in CO_2_ concentration, and the shortened incubation period at the highest temperature avoided invalid measurements once the CO_2_ concentration in the bottle reached saturation. Bottles containing 20 ml of 0.1 M NaOH solution were collected and replaced once every temperature change. Sterile distilled water was regularly added every day throughout the incubation to keep the soil moisture at the water-holding capacity, and the dry weight of each incubated sample was determined at the end of the test. The CO_2_ concentration in each bottle was measured by the alkali absorption method. The soil respiration rate (*R*_s_) of each sample was calculated by the differences in CO_2_ concentration over a certain period of time per unit of dry weight. After incubation, 1 M BaCl_2_ solution was added to the collected NaOH solution, and then 0.1 M HCl was used to titrate NaOH and calculate *R*_s_.

The relationship between temperature and *R*_s_ was described as follows (Davidson et al., 1998; Xu and Qi, 2001):


$$R_{s}=\alpha e^{\beta T}$$

$$Q_{10}=e^{10\beta }$$

where *R*_s_ is the measured soil respiration rate (μg CO_2_ g^−1^ dry soil h^−1^), T is the temperature (°C), and *Q*_10_ is the temperature sensitivity of soil respiration.

### 2.3 Soil biochemical analyses

SOC was determined using the K_2_CrO_7_-H_2_SO_4_ oxidation method (Dai et al., 2021). TN was measured using the Kjeldahl method (Zeng et al., 2016). The MBC and MBN contents were determined by the chloroform fumigation-extraction method with a total organic carbon analyzer (TOC-VCSH, Shimadzu, Japan) described in Vance et al. (1987) and Brookes et al. (1985), respectively, with conversion factors K_C_ of 0.38 for MBC and K_N_ of 0.45 for MBN. The organic carbon in the unfumigated soil extracts was deemed to be DOC. The soil nitrate $\left({\text{NO}}_{3}^{-}-\text{N}\right)$ and ammonium $\left({\text{NH}}_{4}^{+}-\text{N}\right)$ nitrogen were extracted with KCl (l mol L^−1^) and determined by colorimetry using a Bran & Luebbe II AutoAnalyser. N_min_ was the sum of nitrate $\left({\text{NO}}_{3}^{-}-\text{N}\right)$ and ammonium nitrogen $\left({\text{NH}}_{4}^{+}-\text{N}\right)$ . The soil C:N ratio (C/N) was the ratio of DOC to N_min_. Soil pH was determined with a digital pH metre (Woonsocket, RI, USA) using a soil-to-water ratio of 1:2.5 (w/v).

The activities of β-1,4-xylosidase, β-1,4-glucosidase, β-D-cellobiohydrolase and polyphenol oxidase were measured fluorometrically using a 200 μM solution of substrates labelled with 4-methylumbelliferone (MUB), according to the method outlined in Saiya-Cork et al. (2002) and modified by Du et al. (2021). One gram of fresh soil from each treatment was added to 125 ml of 50 mM buffer, and the soil suspension was homogenized in a constant temperature shaker for 2 h. The prepared suspensions continued to be stirred, and then 200-μl aliquots were dispensed into 96-well microplates. Fifty microliters of 200 μM substrate solution were added to 200 μl of the sample suspensions for each sample well; 50 μl of buffer was added to 200 μl of sample suspensions for the blank wells; 50 μl of standard (10 μM 4-methylumbelliferone-MUB) was added to 200 μl of sample suspension for each quench well; 50 μl of substrate solution was added to 200 μl of buffer for the negative control wells; 50 μl of standard was added to 200 μl of buffer for the negative control wells; and 50 μl of standard was added to 200 μl of acetate buffer for the reference standard wells. The prepared plates were incubated in the dark at 25°C for 4 h. To stop the reaction, 50 μl of 0.5 M NaOH was added to each well after incubation. The fluorescence was measured using a microplate reader (SpectraMax Gemini, Molecular Devices, CA, USA) at excitation and emission wavelengths of 365 and 450 nm, respectively. Those activities were corrected for quench and negative controls and are expressed in units of nmol activity per hour per gram of dry soil (μmol d^−1^ g^−1^).

In the harvest stage, two representative quadrats (4 m×1 m) were selected for each plot to determine the economic yield. That is, 5 holes with plants featuring similar growth were selected to determine their podding yields. Roots from plants were separated from soils by soaking in water, nodules on the root were stripped by hand, and both were air-dried for less than two days to determine their fresh weights.

### 2.4 High-throughput sequencing and bioinformatics analysis

Soil DNA was extracted using an E.Z.N.A.^®^ soil DNA kit (Omega Biotek, Norcross, GA, U.S.). The quality of DNA extraction was detected by 1% agarose gel electrophoresis, and the concentration and purity of DNA were evaluated with a NanoDrop 2000 spectrophotometer. The bacterial 16S rRNA gene fragments (V3-V4 region) were amplified using the primers 338F (5′-ACTCCTACGGGAGGCAGCAG-3′) and 806R (5’-GGACTACHVGGGTWTCTAAT-3′), and the fungal ITS1 genes were amplified using primers 1737F (5′-TCCGTAGGTGAACCTGCGG-3′) and 2043R (5′-GCTGCGTTCTTCATCGATGC-3′) (Caporaso et al., 2011). The PCR products were purified from a 2% agarose gel with an AxyPrep DNA Gel Extraction Kit (Axygen Biosciences, Union City, CA, USA) and quantified with a Quantus™ Fluorometer (Promega, USA). Sequencing libraries were constructed with the NEXTFLEX Rapid DNA-Seq Kit. Sequencing was performed using the MiSeq PE300 platform provided by the Illumina corporation.

Raw sequences were processed with QIIME, and chimaeras were detected using UCHIME (Magoč and Salzberg, 2011). After quality filtering and removal of chimeric sequences, all remaining high-quality sequences were clustered into operational taxonomic units (OTUs) at 97% similarity using Usearch software (version 7.0 http://drive5.com/uparse/). Low-abundance OTUs were eliminated from the OTU table if they did not present a total of at least two counts across all samples in the experiment. Taxonomic assignment was conducted using the RDP classifier (http://rdp.cme.msu.edu/) after comparison of each read to the SILVA database (SSU132), with a threshold of 70%. Each sample was rarefied to the same number of reads (74,333 and 80,225 sequences). Sequence analysis was performed by the UPARSE software package using the UPARSE-OTU and UPARSE-OTUref algorithms (Edgar, 2013). Alpha diversity indices, including the Chao1 estimator of richness and Shannon’s diversity index, were generated based on the obtained OTUs. Raw sequence data have been submitted to the National Center for Biotechnology Information (NCBI Bethesda, MD, USA) sequence read archive (SRA) database under accession number PRJNA907179.

### 2.5 Statistical analysis

Differences in soil and plant properties, enzyme activities, microbial properties, *R*_s_ and *Q*_10_ values among the different treatments (mean ± SD, *n*=3) were subjected to ANOVA, followed by the LSD test for *post hoc* comparisons of means at the significance level of 0.05. Pearson correlation analyses were used to identify the relationships between microbial properties and environmental factors, between microbial properties and *R*_s_ and *Q*_10_, and between microbial properties and enzyme activities. The statistical analyses were performed using SPSS 20.0 software for Windows (SPSS Inc., Chicago, USA). Principal coordinate analysis (PCoA) and ADONIS analysis at the OTU level were conducted to identify dissimilarities in the microbial community structures in samples. Redundancy analysis (RDA) at the OTU level was used to identify the most important environmental variables influencing microbial communities. PCoA, ADONIS and RDA were performed with R software version 4.0.3 with the ‘vegan’ package (Team, R.C, 2012). The figures were generated using Sigmaplot 12.5 software (Systat Software Inc., San Jose, CA, USA).

## 3 Results

### 3.1 Changes in soil and plant properties

N fertilization significantly changed soil properties (**Table 1**). Compared with N0, SOC was significantly decreased at N120 (by 8.5%) and N180 (by 7.1%), and DOC was slightly reduced at N60 (by 19.8%) and N120 (by 19.9%), while it was significantly decreased at N180 by 33.7%. The ${\text{NO}}_{3}^{-}-\text{N}$ , as well N_min_, was significantly increased at N120 and N180 by 195.5% and 319.3%, respectively, while ${\text{NH}}_{4}^{+}-\text{N}$ n and TN were unchanged with fertilization. As a result, the mean C/N was significantly reduced from 3.2 at N0 to 1.5, 1.0 and 0.5 at N60, N120 and N180, respectively. MBC was significantly depressed regardless of N application rates, and MBN was significantly reduced only at N120 and N180. All activities of carbon-acquiring enzymes were significantly affected except β-1,4-xylosidase activities. Compared to N0, the activities of β-1,4-glucosidase were significantly decreased by 40.7%, 49.8% and 57.9%, respectively. Polyphenol oxidase activities also significantly decreased by 28.3%, 28.2% and 24.5%, respectively. In contrast, the activity of β-D-cellobiohydrolase was extremely increased by 107.0% at N120 (**Table 1**). In addition, soil pH was significantly reduced at N180 by 3.8% (*P*<0.05). Regarding plant properties, the average root biomass was significantly increased by 61.5% at N120 compared with N0 (3.20 ± 0.09 g plant^−1^). In contrast, the fresh weight of nodules decreased, especially at N180, although the difference was not significant. Unexpectedly, three years of data (**Table 2**) showed that podding yields varied little among treatments (*P*>0.05).

**Table 1:** Soil properties affecting the rhizosphere soil microbial communities sampled in August 2021.

Soil properties	N application rates				
	N0	N60	N120	N180	
pH	5.45 ± 0.05ab	5.39 ± 0.01b	5.54 ± 0.02a	5.24 ± 0.06c
SOC/g kg^−1^	7.07 ± 0.06a	7.07 ± 0.18a	6.47 ± 0.04b	6.57 ± 0.04b
TN/g kg^−1^	0.83 ± 0.02a	0.83 ± 0.02a	0.83 ± 0.04a	0.84 ± 0.01a
DOC/mg kg^−1^	39.86 ± 3.31a	31.98 ± 1.89ab	31.94 ± 6.40ab	26.42 ± 3.04b
N_min_/mg kg^−1^	13.10 ± 1.55c	21.34 ± 0.05c	34.92 ± 5.48b	49.48 ± 0.66a
C/N	3.19 ± 0.65a	1.50 ± 0.09b	1.01 ± 0.36b	0.54 ± 0.07b
MBC/mg kg^−1^	149.09 ± 5.69a	112.37 ± 3.49b	110.25 ± 1.18b	103.94 ± 1.15b
MBN/mg kg^−1^	2.44 ± 0.34a	3.05 ± 0.13a	0.67 ± 0.08b	0.61 ± 0.11b
β-1,4-xylosidase activities/ nmol g^−1^ h^−1^	2.44 ± 0.29a	2.35 ± 0.40a	2.07 ± 0.14a	2.00 ± 0.25a
β-1,4-glucosidase activities/ nmol g^−1^ h^−1^	39.54 ± 4.11a	23.45 ± 0.95b	19.84 ± 3.94b	16.63 ± 1.85b	
β-D-cellobiohydrolase activities/ nmol g^−1^ h^−1^	4.93 ± 0.05b	7.53 ± 1.00b	10.20 ± 1.04a	7.20 ± 0.75b	
polyphenol oxidase activities/ nmol g^−1^ h^−1^	21.98 ± 0.43a	15.75 ± 0.95b	15.78 ± 1.14b	16.59 ± 2.38b	

SOC, soil organic carbon; DOC, dissolved organic carbon; N_min_, soil mineral nitrogen; TN, total nitrogen; C/N, soil carbon: nitrogen ratio; MBC, microbial biomass carbon; MBN, microbial biomass nitrogen. Different lowercase letters denote the differences among treatments at P<0.05, ANOVA ( ± SE, n=3).

**Table 2:** Plant properties affecting the rhizosphere soil microbial communities sampled in August 2021.

Plant properties		N application rates			
		N0	N60	N120	N180
Yields/ kg hm^−2^	2019	5236.37 ± 160.19a	5090.53 ± 498.06a	5375.27 ± 445.56a	5229.43 ± 271.12a
	2020	4052.49 ± 693.62a	4346.62 ± 855.83a	4666.90 ± 367.03a	4810.70 ± 260.72a
	2021	4313.94 ± 321.02a	4693.05 ± 556.64a	4758.41 ± 328.39a	5111.37 ± 210.39a
Root biomass/g plant^−1^		3.20 ± 0.09b	3.35 ± 0.16b	5.17 ± 0.88a	3.53 ± 0.36b
Fresh weight of nodules/mg plant^−1^		144.67 ± 42.16a	107.00 ± 43.27a	123.17 ± 33.37a	37.83 ± 12.03a

Different lowercase letters denote the differences among treatments at *P*<0.05, ANOVA (±SE, *n*=3).

### 3.2 Diversity and relative abundances of the microbial community

**Figure 1:**
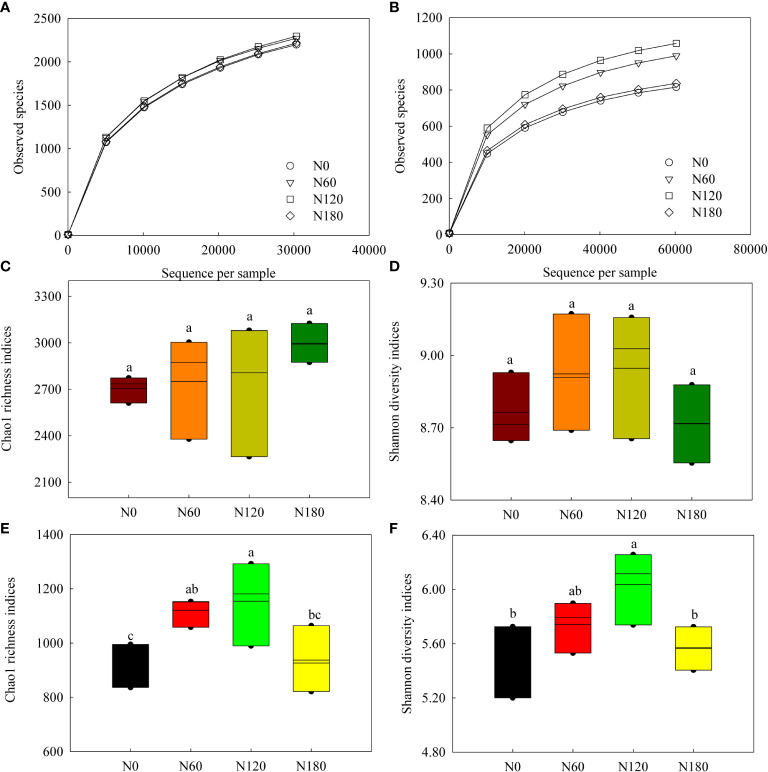
Rarefaction curves based on the observed species **(A, B)**, Chao1 richness **(C, E)** and Shannon diversity **(D, F)** indices of soil bacterial and fungal communities. Different lowercase letters denote the difference among the N levels at *P*<0.05, ANOVA (±SE, *n*=3).

**Figure 2:**
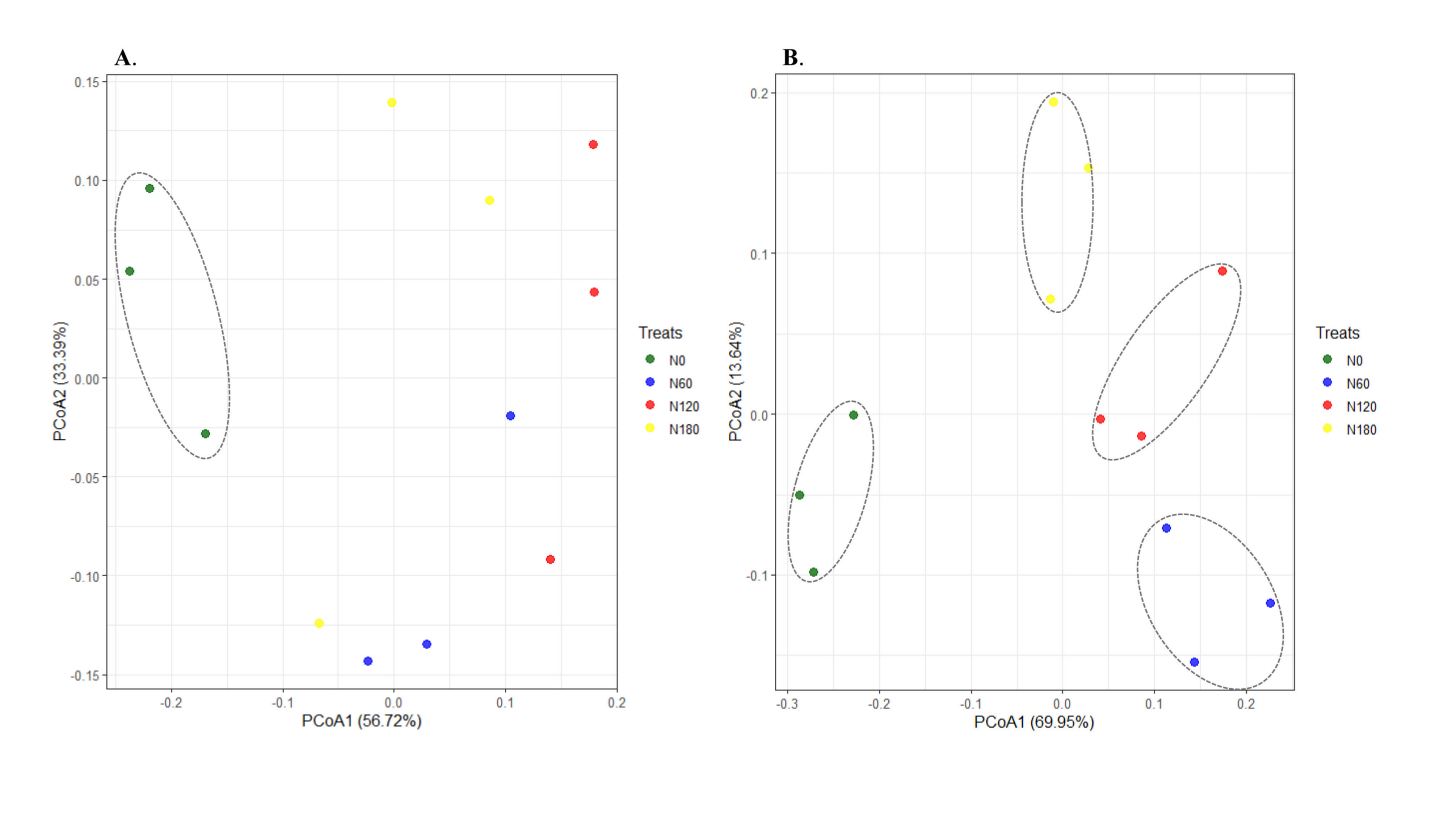
Principal coordinate analysis (PCoA) of soil bacterial **(A)** *P*=0.095 and fungal **(B)** *P*=0.075 communities across the four treatments based on Bray-curtis distance.

In total, 891,994 high-quality sequences (98.6% of the total 904,889) were obtained from the 16S gene sequencing for all soil samples (75,407 to 74,333 sequences per sample), and an average of 2642 OTUs were identified in each sample. In total, 1,965,115 high-quality sequences (98.7% of the total 1,990,567) were obtained from the ITS gene sequencing for all soil samples (70,799 to 87,279 sequences per sample), and an average of 934 OTUs were identified in each sample. For both bacteria and fungi, the rarefaction curves of the rhizosphere soil samples tended to be gentle (**Figures 1A, B**), with coverage over 97%. The alpha-diversity indices of bacterial communities varied little among treatments (**Figures 1C, D**), while both richness and diversity of fungal communities were significantly increased at N60 and N120 compared to N0 (**Figures 1E, F**). Although nonsignificant, PCoA based on Bray-Curtis distance showed that the bacterial OTUs from N60-, N120- and N180- treated soil were more similar to each other but distant from the unfertilized soil (**Figure 2A**, *P*=0.095). Pairwise comparisons showed that the variation in bacterial community structure was significant between N0 and N120 (*R* ^2 =^ 0.60, *P*<0.01). Fungal OTUs subjected to N60, N120 and N180 were well-clustered in different groups and distant from those from N0-treated soil (N0) (**Figure 2B**, *P*=0.075).

**Figure 3:**
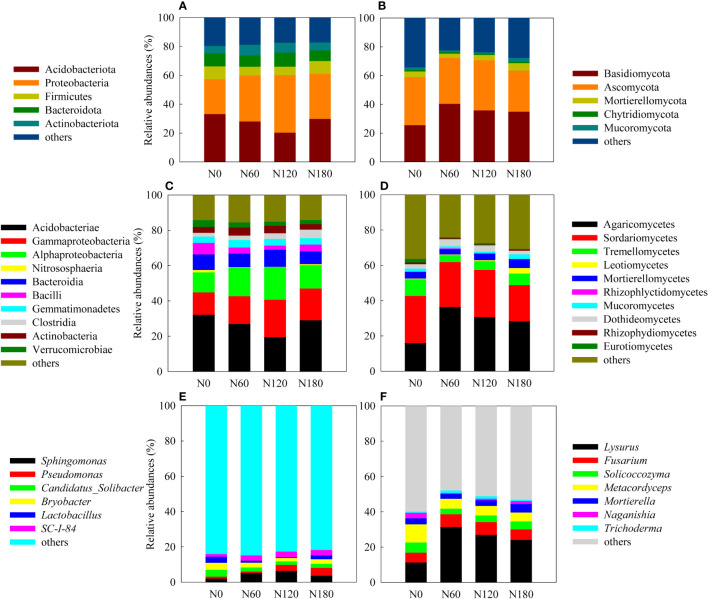
Relative abundances of the dominant bacterial and fungal OTUs at the phylum **(A, B)**, class **(C, D)** and genus **(E, F)** levels.

**Figure 4:**
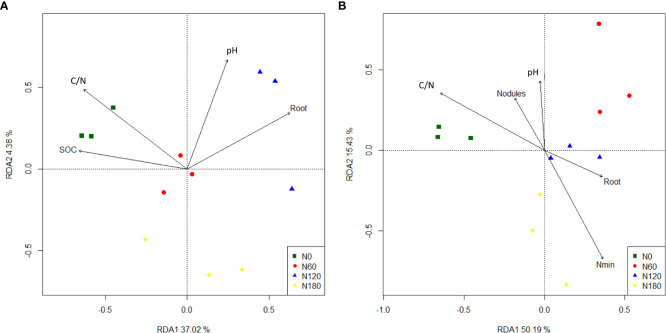
Ordination plots of the redundancy analysis (RDA) to identify relationships between bacterial **(A)** and fungal **(B)** OTUs and environmental variables. SOC, soil organic carbon; DOC, dissolved organic carbon; N_min_, soil mineral nitrogen; C/N, soil carbon: nitrogen ratio; Root: root biomass; Nodules: the fresh weight of nodules.

The most abundant phyla were Acidobacteria at N0 and Proteobacteria at N60, N120 and N180, with the two groups occupying from 57% to 61% of the soil bacteria. Other less abundant phyla included Firmicutes, Bacteroides and Actinobacteria (**Figure 3A**). At the class level, the rhizosphere soil was dominated by Acidobacteria except N120, where the most abundant groups were Gammaproteobacteria and Alphaproteobacteria (**Figure 3C**). More specifically, the relative abundance of Acidobacteria slightly decreased at N60 (by 16.2%, *P*>0.05) and significantly decreased at N120 (by 39.4%, *P*<0.05), with a backup at N180. Due to fertilization, Gammaproteobacteria was promoted especially at N120 (by 66.7%); Alphaproteobacteria was significantly stimulated at both N60 (by 41.9%) and N120 (by 63.7%), and slightly enhanced at N180 (by 16.6%, *P*>0.05). The most abundant order across treatments was Acidobacteriales, followed by Burkholderiales and Sphingomonadales. More specifically, Acidobacteriales was significantly inhibited at N120 compared with N0. In contrast, Burkholderiales and Sphingomonadales were significantly stimulated at both N60 and N120, and Rhizobiales was enhanced only at N120. Correlation results (**Table S1**) showed that the relative abundances of Alphaproteobacteria (order Sphingomonadales and Rhizobiales) and Gammaproteobacteria (order Burkholderiales) were positively correlated with root biomass and β-D-cellobiohydrolase activities, suggesting their copiotrophic strategies; in contrast, the relative abundance of Subgroup_2 was negatively correlated with root biomass and β-D-cellobiohydrolase activities, suggesting their oligotrophic strategies. RDA identified no variable significantly affecting bacterial community composition (**Figure 4A**, *P*>0.05), except root biomass (*R* ^2 =^ 0.39, *P*<0.1).

In rhizosphere soil, the major phyla were Ascomycota at N0 and Basidiomycota at N60, N120 and N180, with the two groups occupying from 59.1% to 72.4% of the soil fungi. Other less abundant phyla included Mortierellomycota, Mucoromycota and Chytridiomycota (**Figure 3B**). At the class level, the rhizosphere soil changed from being dominated by Sordariomycetes at N0 to being dominated by Agaricomycetes at N60, N120 and N180, followed by Sordariomycetes and Tremellomycetes (**Figure 3D**). More specifically, Sordariomycetes seemed equivalent across treatments, with the relative abundances being reduced by 23.1% (*P*<0.05) at N180 than at N0. Compared with N0, the relative abundances of Agaricomycetes were significantly increased by 127.2%, 91.8% and 76.8% at N60, N120 and N180, respectively; in contrast, Tremellomycetes were significantly decreased by 58.8%, 47.2% and 30.6% at N60, N120 and N180, respectively, by N fertilization. Correlation results (**Table S2**) showed that none of the fungal groups correlated with root biomass, while the relative abundances of Agaricomycetes (order Phallales) were positively correlated with β-D-cellobiohydrolase activities throughout the profile, suggesting their important role in decomposing cellulose. The relative abundances of Tremellomycetes were positively correlated with polyphenol oxidase activities but were negatively correlated with β-D-cellobiohydrolase activities. The relative abundances of Eurotiomycetes were positively correlated with β-1,4-glucosidase and polyphenol oxidase activities, suggesting their role in producing enzymes involved in decomposing recalcitrant substrates. Soil pH, DOC, N_min_, C/N, root biomass and nodules explained 87.2% of the total variation in fungal community composition. N_min_ (*R* ^2 =^ 0.65, *P*=0.008) > nodules (*R* ^2 =^ 0.45, *P*=0.050) > C/N (*R* ^2 =^ 0.43, *P*=0.068) was the environmental gradient driving soil fungal community composition and significantly accounted for 52.9% of the total variation (**Figure 4B**, *P*<0.01).

### 3.3 Functional groups of microbial communities

**Figure 5:**
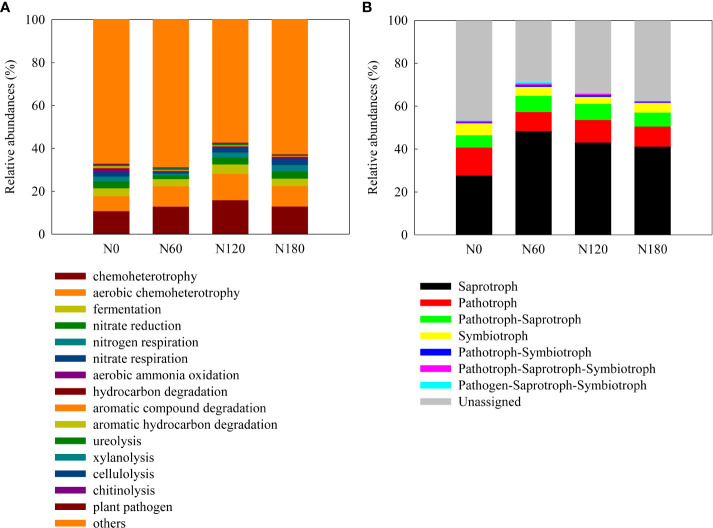
The functionals of bacterial communities and ecological functions of fungal communities explored using FAPROTAX **(A)** and FUNGuild **(B)**.

The composition of bacterial functional groups, inferred from the FARPROTAX database, was significantly affected by N fertilization (**Figure 5A**). Specifically, the OTUs indicating nitrification, nitrate ammonification, aerobic ammonia oxidation, aromatic compound degradation and aromatic hydrocarbon degradation were significantly decreased. The OTUs indicating ureolysis activities were higher at N60 and N120 than at N180, while those indicating plant pathogens were significantly decreased at N60 and N180. The composition of fungal functional groups (trophic modes), inferred from the FUNGuild database (**Figure 5B**), was dominated by saprotrophic fungi, followed by pathogenic fungi and then pathogenic-saprotrophic fungi, with a small percentage of symbiotic fungi. The saprotrophic fungi were significantly increased, while the pathogenic fungi were significantly decreased due to N fertilization. The pathogenic-saprotrophic fungi were unchanged, while the symbiotic fungi decreased slightly at N60 and significantly at N120.

### 3.4 Changes in *R*_s_ and *Q*_10_

**Figure 6:**
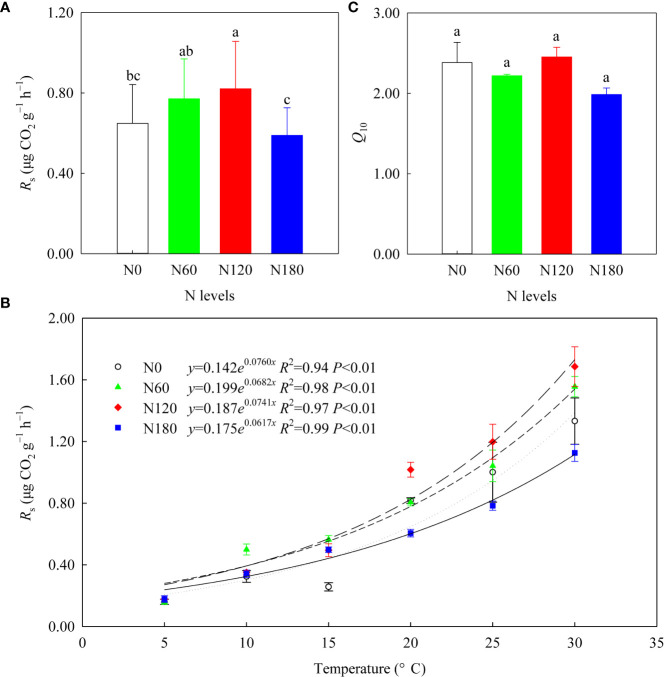
The soil respiration rates (*R*_s_) after averaging six temperatures **(A)**, the exponential growth curves between the mean *R*_s_ and temperature **(B)**, and the mean *Q*_10_ values **(C)** of the four treatments (*n*=3). Different lowercase letters denote the difference among the N levels at *P*<0.05, ANOVA (±SE, *n*=3).

After averaging the six temperatures, the mean *R*_s_ values were slightly and significantly higher at N60 (by 18.9%, *P*>0.05) and N120 (by 26.5%, *P*<0.05) than at N0 (0.56 ± 0.14 μg CO_2_ g^−1^ h^−1^), while that of N180 was slightly lower (by 9.2%, *P*>0.05) than that of N0 (**Table 3**; **Figure 6A**). All the *R*_s_ values increased with increasing temperature, and their correlations could be fitted with exponential growth curves (**Figure 6B**), with *Q*_10_ values numerically varying among treatments (**Figure 6C**, *P*>0.05). The correlation results (**Table S3**) showed that *R*_s_ was positively correlated with soil pH, root biomass and β-D-cellobiohydrolase activities. Furthermore, *R*_s_ was positively correlated with the relative abundances of Actinobacteria, Sphingomonadales, Rhizobiales, Burkholderiaceae, Dothideomycetes (order Pleosporales) and Hypocreaceae (genus *Trichoderma*) (**Tables S1**, **S2**). In contrast, *R*_s_ was negatively correlated with the relative abundances of Subgroup_2 and Tremellomycetes (**Tables S1**, **S2**). In addition, *Q*_10_ was positively correlated with class Sordariomycetes (**Table S2**).

**Table 3:** Soil respiration rate (*R*_s_) and its temperature sensitivity (*Q*_10_) of each treatment under increasing temperature from 5°C to 30°C.

Items		N application rates			
		N0	N60	N120	N180
*R*_s_/μg CO_2_ g^−1^ h^−1^	5°C	0.16 ± 0.02	0.16 ± 0.02	0.18 ± 0.02	0.18 ± 0.02
	10°C	0.32 ± 0.04	0.50 ± 0.04	0.35 ± 0.01	0.34 ± 0.02
	15°C	0.26 ± 0.03	0.56 ± 0.03	0.49 ± 0.04	0.50 ± 0.02
	20°C	0.82 ± 0.02	0.81 ± 0.02	1.02 ± 0.05	0.61 ± 0.02
	25°C	1.00 ± 0.19	1.04 ± 0.10	1.20 ± 0.11	0.78 ± 0.03
	30°C	1.33 ± 0.15	1.56 ± 0.07	1.69 ± 0.13	1.13 ± 0.05
	mean values	0.65 ± 0.19bc	0.77 ± 0.20ab	0.82 ± 0.24a	0.59 ± 0.14c
*Q*_10_		2.38 ± 0.25a	2.22 ± 0.02a	2.45 ± 0.12a	1.99 ± 0.08a

Different lowercase letters denote the difference among the N levels at *P*<0.05, ANOVA (±SE, *n*=3).

## 4 Discussion

### 4.1 N fertilizer enhanced copiotrophic and inhibited oligotrophic bacteria

Initially, at N0, the unfertilized rhizosphere soil was dominated by Acidobacteria (Subgroup_2, **Figure 3C**), which is an acidophilic and oligotrophic bacterium with the ability to decompose cellulose and hemicellulose and fix N (Kalam et al., 2020). N fertilization at a low level (N60) introduced nonsignificantly greater N_min_ (by 63%) into soil but led to significantly decreased C/N (**Table 1**), which suggested improvement of substrate quality. As a result, the oligotrophic Acidobacteria decreased, while members of the copiotrophic Proteobacteria, such as Burkholderiales and Sphingomonadales, were promoted, indicating that a small amount of root-derived C can lead to large variations in microbial community composition. At the normal fertilization rate (N120), the significantly increased N_min_ (by 2.7-fold) induced an enhancement of root biomass (by 61.5%, **Table 1**), which indicated highly stimulated root residues and fresh C inputs from plants. Consequently, Acidobacteria was replaced by copiotrophic groups such as Burkholderiales, Sphingomonadales, and Rhizobiales. Gammaproteobacteria and Alphaproteobacteria have often been associated with copiotrophic bacteria (Liu et al., 2020) and vary in contrast to oligotrophic Acidobacteria. This was confirmed by the negative correlation between Acidobacteria and Gammaproteobacteria (*R* ^2 =^ 0.76, *P*<0.05, data not shown). Furthermore, both Gammaproteobacteria and Alphaproteobacteria were closely related to β-D-cellobiohydrolase activities, thus being highly stimulated at N120, where there is the greatest root biomass. The decreased percentages of Acidobacteria at N60 and N120 could also be ascribed to the high pH of the soil. In contrast, members of Gammaproteobacteria, family Burkholderiaceae, responded positively to the high pH (**Table S4**). Nevertheless, such replacement was not observed at N180. The backup of Acidobacteria at N180 was likely related to the reduced pH. Overfertilization can lead to a significant reduction in soil pH (Zeng et al., 2016; Dong et al., 2021), and excessive N-associated acidification and nutrient imbalance are disadvantageous to root development and yield formation. Simultaneously, the significantly greater N_min_ (by 3.8-fold) resulted in decreased SOC, MBC and DOC, an extremely lower C/N of 0.5 and a deficiency of root-derived C (**Table 1**), indicating an extremely C-limited microbial niche. This, together with the decreased soil pH and suppressed activities of both microbes and plants, led to nearly similar bacterial communities to N0 (**Figures 1**–**3**). In conclusion, the effect of N fertilization on rhizosphere soil bacterial communities seemed to be regulated by the amount of root-derived C inputs (**Figure 4A**).

### 4.2 N fertilization stimulated saprotrophic but depressed pathogenic and symbiotic fungi

The prevalence of Sordariomycetes (genera *Metacordyceps* and *Fusarium*) across treatments (**Figure 3D**) clearly indicated pathogen accumulation due to consecutive monocropping obstacles. Sordariomycetes can cause foot and root rot or bulb rot, thus leading to severe losses in many field crops (Michielse and Rep, 2009). In particular, the genus *Fusarium* has been reported as the pathogen causing root rot in many legumes (Wang et al., 2014). With fertilization, the enhanced Agaricomycetes (genus *Lysurus*) (**Figure 3D**) was likely due to their relevance in cellulose decomposition, confirmed by their positive correlation with β-D-cellobiohydrolase activities (**Table S5**). Moreover, Agaricomycetes may act as saprotrophs (confirmed by their positive correlation, **Table S6**), which was predicted to be enhanced by N fertilization (**Figure 5B**). The preferential growth of saprophytic fungi may be ascribed to the large number of short-lived cells detached from the root cap during crop growth, because saprophytic fungi usually live on dead organic matter (Tisdall and Oades, 1982). In contrast, the suppressed Tremellomycetes and Eurotiomycetes (**Figure 3D**) can be ascribed to their responsibility for the decomposition of recalcitrant C, such as glucose and phenolic oxide (correlation with β-1,4-glucosidase and polyphenol oxidase activities, **Table S5**). Moreover, Tremellomycetes may act as pathotrophs and symbiotrophs, as confirmed by their positive correlation (**Table S6**). The decrease in pathogens with fertilization (**Figure 5B**) was in contrast to that described by Wang et al. (2018), who reported that urea addition increased pathogens. Considering the negative correlation between the relative abundances of Agaricomycetes and Tremellomycetes (*R* ^2 =^ 0.77, *P*<0.05, data not shown), antagonism may explain their opposite variation. Extracts of *Lysurus* mokusion from soil *Lysurus* have inhibitory effects on pathotrophs, by affecting cell membrane permeability and mycelial morphology, and inhibiting the activities of several enzymes associated with growth and pathogenicity (Zhang et al., 2019). Such inhibition may also account for the suppressed pathogenic Sordariomycetes (genus *Metacordyceps*) (**Figure 3F**) and partially explain the reduced β-1,4-glucosidase and polyphenol oxidase activities (**Table 1**). Exceptionally, *Fusarium* was unchanged with fertilization (**Figure 3F**) and may act as a Pathotroph-Saprotroph (positive correlation, **Table S6**). The decreased symbiotrophs were likely due to the reduced dependence of host plants on mycorrhizal fungi for nutrient uptake, as N availability increased (Wang et al., 2018). The impacts of N_min_ and soil C/N on the fungal communities (**Figure 4B**) may be attributed to the disruption of the elemental stoichiometric balance and homeostasis by microorganisms (Sinsabaugh et al., 2009).

### 4.3 Fungal communities were more sensitive than bacterial communities to N fertilization

During the consecutive peanut monoculture, we found divergent responses of bacterial and fungal communities to N fertilization, with that of fungal communities slightly more significant, as evidenced by the alpha diversity, community structure and composition (**Figures 1**–**3**). The main reasons were as follows. First, our results found that N fertilization plausibly exerted an indirect effect on bacterial community composition *via* enhancement of root biomass, which was mainly significant at N120; N fertilization may exert a direct effect on fungal members across application rates, while the effect decreased with increasing application rates. Second, such contrasting responses of bacterial and fungal communities can be ascribed to their different carbon- and nitrogen- acquiring strategies. Several studies using PLFA-^13^C showed that saprophytic fungi outcompete bacteria in utilizing labile plant-derived C in arable ecosystems due to translocation within the hyphal network (Zhang et al., 2022b). Therefore, soil fungi tend to acquire more soil available N to achieve a biomass elemental stoichiometric balance (Cui et al., 2019). This deduction was supported by the functional groups of the bacterial OTUs (**Figure 5A**), which predicted a greater percentage of OTUs involved in N cycling over C cycling. Furthermore, ${\text{NH}}_{4}^{+}-\text{N}$ and ${\text{NO}}_{3}^{-}-\text{N}$ are exclusive N resources for bacteria and fungi in agroecosystems and participate in protein synthesis in bacteria and fungi, respectively (Bottomley et al., 2012). The unchanged ${\text{NH}}_{4}^{+}-\text{N}$ and the highly enhanced ${\text{NO}}_{3}^{-}-\text{N}$ may be responsible for the neutral responses of bacterial alpha diversity and the increased fungal alpha diversity, respectively (**Table 1**; **Figure 1**). The distinct factors driving the variations in bacterial (root biomass) and fungal (N_min_) community composition (**Figure 4**) confirmed their metabolic differentiation and nutrient preferences.

Despite the abovementioned variations, PCoA revealed nonsignificant responses of microbial community structure to N fertilization (*P*>0.05), which rejected our first hypothesis. This was consistent with a previous study which reported that legume plants such as soybean are hardly affected by N-fertilization (Meier et al., 2021). The reason may be that N fertilizer was no longer a limiting factor for rhizosphere microbes due to the existence of the legume-based symbiotic N-fixing system. This symbiotic N-fixing system could effectively utilize C resources from plants, thus being more resistant to environmental changes than a nonsymbiotic N-fixing system. This was reinforced by the legume-based physical protection of soil from microbial utilization (Virk et al., 2022). That is, the input of root exudates and residues, as well as a high combination of microbes/mycorrhizae, improved the soil structure and aggregate composition (Li et al., 2020).

### 4.4 N fertilization affected *R*_s_ mainly through cellulose-associated microbes

**Figure 7:**
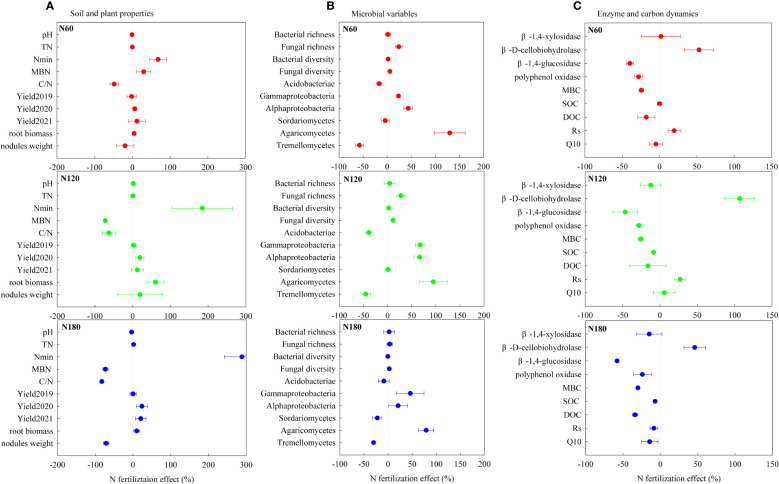
N fertilization induced changes (%) in soil and plant properties **(A)**, microbial variables **(B)** and enzyme and carbon dynamics **(C)** respectively at N60 (A1, B1 and C1), N120 (A2, B2 and C2) and N180 (A3, B3 and C3). SOC, soil organic carbon; DOC, dissolved organic carbon; N_min_, soil mineral nitrogen; TN, total nitrogen; C/N, soil carbon: nitrogen ratio; MBC, microbial biomass carbon; MBN, microbial biomass nitrogen. *R*_s_ and *Q*_10_ represent the soil respiration rate and its temperature sensitivity, respectively.

With N fertilization, the enhanced *R*_s_ at N60 and N120 (**Table 2**; **Figure 6A**) may be a joint result of increased root biomass, β-D-cellobiohydrolase activities, fungal alpha diversity, and alteration of microbial trophic strategies (**Table 1**, **Tables S1**-**S3**; **Figure 7**), which partially supported the second hypothesis. A strong relationship between *R*_s_ and root biomass (**Table S3**) underlines the importance of C input *via* root turnover and root exudations in rhizosphere soil C cycling. Positive correlations between *R*_s_ and Burkholderiales, Sphingomonadales and Rhizobiales (**Table S1**) were likely due to their relevance in accelerating β-D-cellobiohydrolase activities, which directly contributed to *R*_s_ (**Table S3**). The positive correlations between *R*_s_ and Actinobacteria and Dothideomycetes (order Pleosporales) were plausibly related to their contribution to an increase in available C sources through lignin degradation, which would produce a release of simpler compounds to soil (Vida et al., 2016). The positive correlation between *R*_s_ and Hypocreaceae (genus *Trichoderma*) was likely related to their critical role in producing cell wall-degrading enzymes (chitinase, β-1,3-glucanase and cellulase) (Konappa et al., 2022). Although not directly correlated with *R*_s_, Agaricomycetes may also contribute to *R*_s_ through their relevance in cellulose decomposition. Evidence from both microbes and enzymes indicated that the stimulated mineralization of root derived C (mainly cellulose) resulted from the stimulation of microbes able to provide SOC-specific enzymes, in line with previous research (Fontaine et al., 2004).

Initially, at N0, the microbial community composition was dominated by *K*-strategist SOC decomposers (degrading part of cellulose and, mainly, SOC), such as Acidobacteria and Sordariomycetes. At N60, the stimulated *r*-strategist cellulose decomposers (degrading exclusively cellulose), such as Gammaproteobacteria, Alphaproteobacteria, and Agaricomycetes, led to slightly increased *R*_s_ and slightly decreased DOC (**Figure 7A**). As reported, soil C accumulation can be attributed to an increase in C supply to the soil, to a decrease in SOC mineralization to CO_2_, or to some combination of the two (Marshall et al., 2021). The unchanged SOC at N60 relative to N0 could be interpreted as the effect of slightly enhanced C supply being counteracted by that of the slightly increased substrate mineralization. At N120, mineral nutrients could be interpreted as abundant. The *r*-strategist cellulose decomposers and the *K*-strategist SOC decomposers were both greatly stimulated (as evidenced by the decreased SOC), which resulted in dramatically accelerated *R*_s_ and slightly reduced DOC relative to N0 (**Figure 7B**). In the final analysis, the promoted *R*_s_ can be attributed to the triggered mineralization of old C through the input of fresh and easily degradable substrate as a result of the priming effect (Carney et al., 2007). Nevertheless, the reduction in SOC accumulation led us to conclude that the effect of the promoted soil C supply was less than that of the stimulated substrate mineralization. At N180, C limitation concurrently stimulated SOC decomposers (decreased SOC) and constrained cellulose decomposers (slightly decreased *R*_s_). The limited root biomass, microbial diversity, activities and dominant *K*-strategist SOC decomposers jointly resulted in slightly decreased *R*_s_ at N180 (**Figure 7C**). The reduction in SOC decomposition led us to conclude that the decreased soil C accumulation was entirely due to inhibited belowground C allocation. Our results suggested that excessive N fertilization not only stimulated soil CO_2_ emissions but also decreased belowground C allocation by crops and rhizo-C incorporation into microorganisms (MBC) and SOC, in line with the results of Ge et al. (2017).

### 4.5 Neutral response of *Q*_10_ to N fertilization

The unchanged *Q*_10_ (**Table 2**; **Figure 6C**) may be a joint result of increased chemical recalcitrance, improved substrate quality/quantity and enzyme patterns that counteracted each other, which rejected our second hypothesis. Especially for N60, the lower C/N indicates improved substrate quality, in theory, reduced *Q*_10_ (Bosatta and Ågren, 1999). In contrast, the decreased activities of β-1,4-glucosidase and polyphenol oxidase, theoretically indicated reduced degradation of recalcitrant SOC and therefore an increase in *Q*_10_ (Bosatta and Ågren, 1999). Therefore, the slightly reduced *Q*_10_ at N60 compared with N0 was likely the joint result of these two mechanisms that counteracted each other. Specifically, at N120, the extremely stimulated β-D-cellobiohydrolase activities counteracted the suppressed activities of β-1,4-glucosidase and polyphenol oxidase, which in theory reduced *Q*_10_. Nevertheless, the increased substrate availability resulted in the slightly higher *Q*_10_ relative to N0. Moreover, at N180, the slightly lower *Q*_10_ compared with N0 was likely related to microbial C limitation, and to a lesser extent associated with substrate quality. The positive correlation between *Q*_10_ and Sordariomycetes (**Table S2**) verified that *Q*_10_ is linked with a *K*-selected microbial community (Li et al., 2021). In addition, the labile C pool also showed a neutral response to N fertilization, as evidenced by the unchanged β-1,4-xylosidase activities and DOC/SOC (**Table 1**). The contribution of DOC to SOC was less sensitive to N fertilization than that of MBC, which suggested that DOC is in equilibrium with SOC, as DOC may be mainly derived from native SOC rather than the input of photosynthate-C (Lu et al., 2002).

## 5 Conclusions

Under a 5-year continuous peanut monoculture, N fertilization exerted divergent effects on the rhizosphere soil bacterial and fungal communities, both varying with N levels. N fertilization nonsignificantly affected bacterial alpha diversity but increased both richness and diversity of fungal communities. Especially at N120, N fertilization stimulated Alphaproteobacteria and Gammaproteobacteria, which were positively correlated with root biomass and β-D-cellobiohydrolase activities. Regardless of application rates, N fertilization enhanced Agaricomycetes, which were positively correlated with β-D-cellobiohydrolase activities. N fertilization increased *R*_s_ slightly at N60 and significantly at N120 and slightly decreased *R*_s_ at N180 but showed a nonsignificant effect on *Q*_10_. We concluded that N fertilization mediates rhizosphere soil C dynamics mainly by altering cellulose-related microbial communities and enzymes involved in cellulose decomposition. The effect of N fertilization on the rhizosphere soil microbial communities and C dynamics may be regulated by the amount of root-derived C inputs.

This study provides a mechanistic understanding of how rhizosphere soil microbial communities and functions respond to N fertilization in continuous monocropping ecosystems, by relating microbial communities to soil C cycling. The obtained results are of critical significance for the sustainability of such intensive agroecosystems and the accurate assessment of the potential for soil C emissions, especially under global warming.

## Data availability statement

The datasets presented in this study can be found in online repositories. The names of the repository/repositories and accession number(s) can be found below: NCBI accession PRJNA907179.

## Author contributions

ZW: Conceptualization, writing-review & editing. ZT: Methodology, writing-review & editing. TY: Investigation, data management. JZ: Project administration, supervision. YZ: Methodology, investigation. JY: Data curation, validation. YW: Methodology, formal analysis. QS: Funding acquisition, writing-original draft, visualization, writing-review & editing. All authors contributed to the article and approved the submitted version.
