# Supplementary material for: Nitrogen fertilization rates mediate rhizosphere soil carbon emissions of continuous peanut monoculture by altering cellulose-specific microbess
Source: Front Plant Sci. 2023 Mar 3;14:1109860. doi: 10.3389/fpls.2023.1109860 (PMC10021708; doi:10.3389/fpls.2023.1109860)
Supplement: Supplementary file 1 [file Table_1.docx]

Supplementary Material

# Table S1 Correlations between bacterial alpha-diversity, community composition and *R*_s_, *Q*_10_, environmental variables

| Correlations | *R*_s_/  μg CO_2_ g^−1^ h^−1^ | *Q*_10_ | pH | SOC/  g kg^−1^ | TN/  g kg^−1^ | DOC/  g kg^−1^ | N_min_/  g kg^−1^ | Yields/  kg hm^−1^ | Root biomass/  g plant^−1^ | Fresh weight of nodules/  mg plant^−1^ |
| --- | --- | --- | --- | --- | --- | --- | --- | --- | --- | --- |
| Chao1 richness indices | -0.05 | 0.14 | -0.01 | -0.08 | -0.58^*^ | 0.21 | -0.09 | -0.21 | -0.13 | 0.31 |
| Shannon diversity indices | 0.36 | 0.49 | 0.30 | 0.01 | -0.66^*^ | 0.19 | -0.24 | -0.37 | -0.07 | 0.24 |
| *Phylum* |  |  |  |  |  |  |  |  |  |  |
| Acidobacteriota | -0.56 | -0.39 | -0.41 | 0.44 | 0.31 | 0.07 | -0.21 | 0.12 | -0.46 | -0.08 |
| Proteobacteria | 0.61^*^ | 0.15 | 0.31 | -0.60^*^ | -0.02 | -0.33 | 0.45 | 0.11 | 0.61^*^ | -0.01 |
| Firmicutes | -0.55 | 0.22 | -0.30 | 0.27 | -0.38 | 0.19 | -0.05 | -0.40 | -0.59^*^ | -0.27 |
| Bacteroidota | 0.20 | 0.39 | 0.57 | -0.10 | -0.37 | 0.32 | -0.27 | -0.19 | 0.31 | 0.46 |
| Actinobacteriota | 0.64^*^ | 0.22 | 0.24 | -0.14 | -0.32 | -0.08 | -0.06 | -0.10 | 0.16 | 0.16 |
| *Class* |  |  |  |  |  |  |  |  |  |  |
| Acidobacteriae | -0.58 | -0.39 | -0.42 | 0.43 | 0.30 | 0.07 | -0.20 | 0.11 | -0.46 | -0.09 |
| Gammaproteobacteria | 0.30 | 0.17 | 0.12 | -0.56 | -0.18 | -0.29 | 0.51 | -0.05 | 0.45 | -0.16 |
| Alphaproteobacteria | 0.80^**^ | 0.08 | 0.45 | -0.43 | 0.19 | -0.26 | 0.22 | 0.27 | 0.61* | 0.18 |
| Nitrososphaeria | -0.57 | -0.11 | -0.14 | 0.32 | -0.03 | 0.34 | -0.38 | 0.02 | -0.32 | 0.21 |
| Bacteroidia | 0.21 | 0.39 | 0.57 | -0.10 | -0.37 | 0.32 | -0.27 | -0.19 | 0.31 | 0.46 |
| Bacilli | -0.47 | 0.27 | -0.22 | 0.60^*^ | -0.23 | 0.49 | -0.42 | -0.53 | -0.62^*^ | -0.19 |
| Gemmatimonadetes | 0.26 | -0.29 | -0.01 | 0.04 | 0.48 | -0.17 | -0.02 | 0.15 | 0.02 | 0.03 |
| Clostridia | -0.30 | -0.10 | -0.21 | -0.32 | -0.22 | -0.35 | 0.45 | 0.08 | -0.16 | -0.17 |
| Actinobacteria | 0.60^*^ | 0.39 | 0.30 | 0.05 | -0.49 | 0.16 | -0.26 | -0.23 | 0.06 | 0.17 |
| Verrucomicrobiae | -0.29 | -0.03 | 0.18 | 0.60* | 0.08 | 0.46 | -0.69^*^ | 0.03 | -0.35 | 0.37 |
| *Order* |  |  |  |  |  |  |  |  |  |  |
| Acidobacteriales | -0.56 | -0.48 | -0.48 | 0.30 | 0.39 | -0.09 | -0.03 | 0.18 | -0.38 | -0.21 |
| Burkholderiales | 0.78^**^ | 0.28 | 0.35 | -0.22 | -0.17 | -0.12 | 0.09 | -0.07 | 0.41 | 0.03 |
| Nitrosotaleales | -0.59^*^ | -0.15 | -0.22 | 0.30 | -0.03 | 0.31 | -0.32 | 0.04 | -0.32 | 0.14 |
| Xanthomonadales | 0.12 | -0.03 | 0.20 | -0.44 | 0.39 | -0.26 | 0.32 | 0.19 | 0.44 | -0.03 |
| Sphingomonadales | 0.81^**^ | 0.13 | 0.44 | -0.44 | 0.16 | -0.30 | 0.26 | 0.26 | 0.61^*^ | 0.10 |
| Rhizobiales | 0.66^*^ | -0.06 | 0.32 | -0.51 | 0.29 | -0.38 | 0.39 | 0.26 | 0.66^*^ | 0.09 |
| Gemmatimonadales | 0.26 | -0.29 | -0.01 | 0.04 | 0.48 | -0.17 | -0.02 | 0.15 | 0.02 | 0.03 |
| Pseudomonadales | -0.21 | -0.11 | -0.12 | -0.41 | 0.03 | -0.33 | 0.53 | 0.05 | 0.21 | -0.20 |
| Chitinophagales | 0.49 | 0.37 | 0.70^*^ | -0.19 | 0.00 | 0.36 | -0.34 | 0.12 | 0.63^*^ | 0.57^*^ |
| Lactobacillales | -0.47 | 0.21 | -0.26 | 0.49 | -0.10 | 0.37 | -0.32 | -0.21 | -0.61^*^ | -0.25 |
| *Family* |  |  |  |  |  |  |  |  |  |  |
| Nitrosotaleaceae | -0.59^*^ | -0.15 | -0.22 | 0.30 | -0.03 | 0.31 | -0.32 | 0.04 | -0.32 | 0.14 |
| Rhodanobacteraceae | 0.03 | -0.11 | 0.16 | -0.37 | 0.44 | -0.23 | 0.29 | 0.19 | 0.37 | -0.03 |
| Sphingomonadaceae | 0.81^**^ | 0.13 | 0.44 | -0.44 | 0.16 | -0.30 | 0.26 | 0.26 | 0.61^*^ | 0.10 |
| Gemmatimonadaceae | 0.26 | -0.29 | -0.01 | 0.04 | 0.48 | -0.17 | -0.02 | 0.15 | 0.02 | 0.03 |
| Pseudomonadaceae | -0.21 | -0.15 | -0.15 | -0.43 | 0.04 | -0.35 | 0.57 | 0.08 | 0.23 | -0.22 |
| Chitinophagaceae | 0.48 | 0.37 | 0.70^*^ | -0.19 | 0.01 | 0.36 | -0.33 | 0.13 | 0.63^*^ | 0.58 |
| SC-I-84 | 0.37 | -0.27 | -0.05 | -0.46 | 0.32 | -0.62^*^ | 0.65^*^ | 0.28 | 0.33 | -0.32 |
| Burkholderiaceae | 0.65^*^ | 0.45 | 0.71^**^ | -0.17 | -0.07 | 0.34 | -0.30 | -0.10 | 0.55 | 0.49 |
| Lactobacillaceae | -0.51 | 0.05 | -0.30 | 0.42 | 0.08 | 0.27 | -0.24 | -0.02 | -0.58^*^ | -0.22 |
| Subgroup_2 | -0.67^*^ | -0.32 | -0.61^*^ | 0.57 | -0.01 | 0.15 | -0.23 | -0.04 | -0.68^*^ | -0.17 |
| *Genus* |  |  |  |  |  |  |  |  |  |  |
| *Sphingomonas* | 0.77^**^ | 0.06 | 0.36 | -0.41 | 0.21 | -0.34 | 0.30 | 0.29 | 0.58^*^ | 0.03 |
| *Pseudomonas* | -0.20 | -0.15 | -0.14 | -0.44 | 0.05 | -0.37 | 0.57 | 0.09 | 0.23 | -0.21 |
| *Candidatus_Solibacter* | -0.37 | -0.02 | 0.07 | 0.53 | 0.12 | 0.50 | -0.62^*^ | -0.10 | -0.37 | 0.43 |
| *Bryobacter* | -0.43 | -0.04 | -0.09 | 0.56 | 0.06 | 0.43 | -0.57 | -0.01 | -0.44 | 0.25 |
| *Lactobacillus* | -0.51 | 0.05 | -0.30 | 0.42 | 0.07 | 0.28 | -0.24 | -0.02 | -0.58^*^ | -0.21 |
| *SC-I-84* | 0.37 | -0.27 | -0.05 | -0.46 | 0.32 | -0.62^*^ | 0.65^*^ | 0.28 | 0.33 | -0.32 |

Note: SOC, soil organic carbon; DOC, dissolved organic carbon; N_min_, soil mineral nitrogen; TN, total nitrogen; C/N, soil carbon: nitrogen ratio. ^*^indicate significant correlation at *P*<0.05, ^**^ indicate significant correlation at *P*<0.01.

# Table S2 Correlations between fungal alpha-diversity, community composition and *R*_s_, *Q*_10_, environmental variables

| Correlations | *R*_s_/  μg CO_2_ g^−1^ h^−1^ | *Q*_10_ | pH | SOC/  g kg^−1^ | TN/  g kg^−1^ | DOC/  g kg^−1^ | N_min_/  g kg^−1^ | Yields/  kg hm^−1^ | Root biomass/  g plant^−1^ | Fresh weight of nodules/  mg plant^−1^ |
| --- | --- | --- | --- | --- | --- | --- | --- | --- | --- | --- |
| Chao1 richness indices | 0.59^*^ | 0.24 | 0.28 | -0.08 | -0.13 | -0.10 | -0.06 | -0.17 | 0.04 | 0.06 |
| Shannon diversity indices | 0.53 | 0.38 | 0.33 | -0.29 | -0.18 | -0.15 | 0.14 | -0.12 | 0.21 | -0.11 |
| *Phylum* |  |  |  |  |  |  |  |  |  |  |
| Basidiomycota | 0.50 | -0.29 | -0.08 | -0.35 | 0.16 | -0.49 | 0.37 | 0.36 | 0.43 | -0.06 |
| Ascomycota | 0.51 | 0.74^**^ | 0.67^*^ | 0.27 | -0.04 | 0.25 | -0.31 | -0.36 | 0.27 | 0.03 |
| Mortierellomycota | -0.66^*^ | -0.21 | -0.60^*^ | -0.27 | -0.14 | 0.01 | 0.48 | 0.09 | -0.20 | -0.47 |
| Chytridiomycota | -0.09 | -0.40 | 0.21 | -0.17 | 0.36 | -0.41 | 0.01 | 0.42 | 0.06 | 0.26 |
| Mucoromycota | -0.85^**^ | -0.51 | -0.49 | -0.03 | 0.28 | -0.25 | 0.41 | 0.38 | -0.44 | -0.45 |
| *Class* |  |  |  |  |  |  |  |  |  |  |
| Agaricomycetes | 0.56 | -0.21 | -0.05 | -0.35 | 0.11 | -0.51 | 0.37 | 0.30 | 0.43 | -0.11 |
| Sordariomycetes | 0.43 | 0.57^*^ | 0.64^*^ | 0.43 | -0.09 | 0.41 | -0.60^*^ | -0.43 | -0.07 | 0.31 |
| Tremellomycetes | -0.66^*^ | -0.06 | -0.05 | 0.30 | 0.05 | 0.49 | -0.32 | -0.05 | -0.35 | 0.22 |
| Leotiomycetes | -0.41 | -0.28 | -0.57 | -0.28 | -0.05 | -0.13 | 0.54 | 0.19 | 0.18 | -0.44 |
| Mortierellomycetes | -0.66^*^ | -0.21 | -0.64^*^ | -0.27 | -0.14 | 0.01 | 0.48 | 0.09 | -0.20 | -0.47 |
| Rhizophlyctidomycetes | 0.46 | -0.07 | -0.04 | -0.17 | -0.17 | -0.23 | -0.09 | -0.01 | 0.12 | 0.38 |
| Mucoromycetes | -0.87^**^ | -0.51 | -0.51 | 0.00 | 0.25 | -0.21 | 0.37 | 0.36 | -0.49 | -0.43 |
| Dothideomycetes | 0.76^**^ | 0.42 | 0.40 | 0.12 | -0.33 | 0.04 | -0.35 | -0.28 | 0.09 | 0.23 |
| Rhizophydiomycetes | -0.38 | -0.35 | 0.21 | 0.06 | 0.40 | -0.17 | -0.06 | 0.43 | -0.08 | 0.07 |
| Eurotiomycetes | -0.09 | 0.48 | 0.37 | 0.54 | -0.13 | 0.59^*^ | -0.75^**^ | -0.36 | -0.28 | 0.41 |
| *Order* |  |  |  |  |  |  |  |  |  |  |
| Phallales | 0.56 | -0.23 | -0.02 | -0.38 | 0.17 | -0.56 | 0.42 | 0.35 | 0.44 | -0.14 |
| Hypocreales | 0.21 | 0.43 | 0.49 | 0.64^*^ | -0.11 | 0.57 | -0.77^**^ | -0.48 | -0.27 | 0.36 |
| Filobasidiales | -0.66^*^ | -0.04 | -0.04 | 0.30 | 0.05 | 0.49 | -0.32 | -0.05 | -0.35 | 0.22 |
| Sordariales | -0.14 | -0.13 | -0.28 | -0.28 | 0.04 | -0.50 | 0.55 | 0.27 | -0.24 | -0.37 |
| Agaricales | 0.02 | 0.38 | 0.25 | -0.04 | 0.23 | 0.12 | 0.01 | -0.15 | 0.28 | -0.08 |
| Mortierellales | -0.66^*^ | -0.21 | -0.60^*^ | -0.27 | -0.14 | 0.01 | 0.48 | 0.09 | -0.20 | -0.47 |
| Mucorales | -0.87^**^ | -0.51 | -0.51 | 0.00 | 0.25 | -0.21 | 0.37 | 0.36 | -0.49 | -0.43 |
| Melanosporales | 0.31 | 0.25 | 0.17 | 0.13 | -0.14 | 0.32 | -0.41 | -0.27 | 0.32 | 0.38 |
| Pleosporales | 0.77^**^ | 0.46 | 0.43 | -0.03 | -0.31 | 0.05 | -0.21 | -0.16 | 0.21 | 0.15 |
| Erysiphales | -0.38 | -0.26 | -0.58 | -0.20 | -0.14 | 0.00 | 0.38 | 0.10 | 0.12 | -0.35 |
| *Family* |  |  |  |  |  |  |  |  |  |  |
| Phallaceae | 0.56 | -0.23 | -0.02 | -0.38 | 0.17 | -0.56 | 0.42 | 0.35 | 0.44 | -0.14 |
| Nectriaceae | 0.37 | 0.24 | 0.28 | 0.24 | -0.20 | 0.05 | -0.20 | -0.34 | -0.16 | 0.03 |
| Chaetomiaceae | -0.23 | -0.25 | -0.29 | -0.08 | 0.16 | -0.49 | 0.46 | 0.24 | -0.37 | -0.38 |
| Piskurozymaceae | -0.66^*^ | -0.06 | -0.06 | 0.28 | 0.07 | 0.47 | -0.27 | -0.02 | -0.36 | 0.16 |
| Clavicipitaceae | -0.26 | 0.16 | 0.23 | 0.54 | 0.10 | 0.53 | -0.63^*^ | -0.27 | -0.29 | 0.32 |
| Mortierellaceae | -0.67^*^ | -0.20 | -0.60^*^ | -0.28 | -0.14 | 0.02 | 0.48 | 0.10 | -0.19 | -0.47 |
| Filobasidiaceae | -0.64^*^ | -0.03 | -0.02 | 0.30 | 0.01 | 0.50 | -0.37 | -0.09 | -0.33 | 0.27 |
| Mucoraceae | -0.83^**^ | -0.37 | -0.37 | 0.19 | 0.23 | -0.02 | 0.13 | 0.33 | -0.54 | -0.34 |
| Hypocreaceae | 0.76^**^ | 0.18 | 0.47 | 0.07 | 0.12 | -0.13 | -0.11 | -0.03 | 0.39 | 0.00 |
| *Genus* |  |  |  |  |  |  |  |  |  |  |
| *Lysurus* | 0.56 | -0.23 | -0.02 | -0.38 | 0.17 | -0.56 | 0.42 | 0.35 | 0.44 | -0.14 |
| *Fusarium* | 0.30 | 0.22 | 0.26 | 0.15 | -0.17 | -0.04 | -0.04 | -0.22 | -0.11 | -0.08 |
| *Solicoccozyma* | -0.66^*^ | -0.06 | -0.06 | 0.29 | 0.07 | 0.47 | -0.28 | -0.02 | -0.36 | 0.17 |
| *Metacordyceps* | -0.27 | 0.16 | 0.22 | 0.54 | 0.09 | 0.55 | -0.64^*^ | -0.28 | -0.30 | 0.33 |
| *Naganishia* | -0.65^*^ | -0.03 | -0.02 | 0.30 | 0.01 | 0.49 | -0.36 | -0.08 | -0.33 | 0.26 |
| *Mortierella* | -0.73^**^ | -0.28 | -0.68^*^ | -0.25 | -0.14 | -0.01 | 0.49 | 0.11 | -0.25 | -0.49 |

Note: SOC, soil organic carbon; DOC, dissolved organic carbon; N_min_, soil mineral nitrogen; TN, total nitrogen; C/N, soil carbon: nitrogen ratio. ^*^indicate significant correlation at *P*<0.05, ^**^ indicate significant correlation at *P*<0.01.

# Table S3 Correlations between *R*_s_, *Q*_10_, microbial biomass, enzyme activities and environmental variables

| Correlations | *R*_s_/  μg CO_2_ g^−1^ h^−1^ | *Q*_10_ | MBC/  g kg^−1^ | β-1,4-xylosidase /  nmol g^−1^ h^−1^ | β-1,4-glucosidase/  nmol g^−1^ h^−1^ | β-D-cellobiohydrolase/  nmol g^−1^ h^−1^ | polyphenol oxidase/  nmol g^−1^ h^−1^ |
| --- | --- | --- | --- | --- | --- | --- | --- |
| *R*_s_/μg CO_2_ g^−1^ h^−1^ | 1.00 | 0.53 | -0.26 | 0.11 | -0.08 | 0.60^*^ | -0.40 |
| *Q*_10_ | 0.53 | 1.00 | 0.18 | 0.01 | 0.46 | 0.20 | 0.11 |
| pH | 0.59^*^ | 0.46 | 0.36 | 0.17 | 0.33 | 0.17 | -0.08 |
| SOC/g kg^−1^ | -0.14 | 0.11 | 0.60^*^ | 0.04 | 0.57^*^ | -0.71^**^ | 0.28 |
| TN/g kg^−1^ | -0.07 | -0.54 | 0.07 | -0.15 | -0.09 | -0.33 | -0.22 |
| DOC/g kg^−1^ | 0.00 | 0.52 | 0.57 | 0.03 | 0.47 | -0.09 | 0.54 |
| N_min_/g kg^−1^ | -0.16 | -0.39 | -0.75^**^ | -0.33 | -0.67^*^ | 0.35 | -0.50 |
| Yields/kg hm^−2^ | -0.05 | -0.26 | -0.13 | 0.04 | -0.27 | 0.20 | 0.25 |
| Root biomass/g plant^−1^ | 0.60^*^ | 0.23 | -0.30 | 0.11 | -0.22 | 0.66^*^ | -0.35 |
| Fresh weight of nodules/mg plant^−1^ | 0.25 | 0.11 | 0.47 | 0.46 | 0.22 | 0.10 | 0.20 |

Note: SOC, soil organic carbon; DOC, dissolved organic carbon; N_min_, soil mineral nitrogen; TN, total nitrogen; C/N, soil carbon: nitrogen ratio; MBC, microbial biomass carbon. ^*^indicate significant correlation at *P*<0.05, ^**^ indicate significant correlation at *P*<0.01.

# Table S4 Correlations between bacterial alpha-diversity, community composition and enzyme activities

| Correlations | MBC/  g kg^−1^ | β-1,4-xylosidase/  nmol g^−1^ h^−1^ | β-1,4-glucosidase/  nmol g^−1^ h^−1^ | β-D-cellobiohydrolase/  nmol g^−1^ h^−1^ | polyphenol oxidase/  nmol g^−1^ h^−1^ |
| --- | --- | --- | --- | --- | --- |
| Chao1 richness indices | -0.11 | 0.14 | -0.30 | 0.26 | -0.22 |
| Shannon diversity indices | -0.22 | -0.02 | -0.12 | 0.39 | -0.24 |
| *Phylum* |  |  |  |  |  |
| Acidobacteriota | 0.51 | 0.25 | 0.48 | -0.73^**^ | 0.54 |
| Proteobacteria | -0.69^*^ | -0.26 | -0.71^**^ | 0.79^**^ | -0.76^**^ |
| Firmicutes | 0.16 | -0.35 | 0.19 | -0.47 | 0.16 |
| Bacteroidota | 0.24 | 0.34 | 0.12 | 0.35 | 0.08 |
| Actinobacteriota | -0.49 | -0.01 | -0.21 | 0.57 | -0.32 |
| *Class* |  |  |  |  |  |
| Acidobacteriae | 0.51 | 0.26 | 0.48 | -0.72^**^ | 0.55 |
| Gammaproteobacteria | -0.62^*^ | -0.25 | -0.66^*^ | 0.64^*^ | -0.64^*^ |
| Alphaproteobacteria | -0.53 | -0.18 | -0.52 | 0.70^*^ | -0.64^*^ |
| Nitrososphaeria | 0.74^**^ | 0.57 | 0.60^*^ | -0.54 | 0.73^**^ |
| Bacteroidia | 0.23 | 0.34 | 0.12 | 0.36 | 0.08 |
| Bacilli | 0.52 | -0.35 | 0.58^*^ | -0.69^*^ | 0.59^*^ |
| Gemmatimonadetes | -0.10 | -0.29 | -0.10 | -0.06 | -0.04 |
| Clostridia | -0.39 | -0.11 | -0.46 | 0.10 | -0.49 |
| Actinobacteria | -0.32 | -0.04 | -0.05 | 0.50 | -0.16 |
| Verrucomicrobiae | 0.86^**^ | 0.46 | 0.67^*^ | -0.57 | 0.68^*^ |
| *Order* |  |  |  |  |  |
| Acidobacteriales | 0.35 | 0.21 | 0.36 | -0.63^*^ | 0.47 |
| Burkholderiales | -0.60^*^ | -0.25 | -0.44 | 0.71^*^ | -0.57 |
| Nitrosotaleales | 0.67^*^ | 0.56 | 0.53 | -0.51 | 0.69^*^ |
| Xanthomonadales | -0.04 | -0.08 | 0.14 | 0.11 | 0.11 |
| Sphingomonadales | -0.58^*^ | -0.22 | -0.57 | 0.72^**^ | -0.70^*^ |
| Rhizobiales | -0.58^*^ | -0.16 | -0.57 | 0.65^*^ | -0.67^*^ |
| Gemmatimonadales | -0.10 | -0.29 | -0.10 | -0.06 | -0.04 |
| Pseudomonadales | -0.29 | -0.06 | -0.49 | 0.17 | -0.45 |
| Chitinophagales | 0.39 | 0.47 | 0.33 | 0.39 | 0.29 |
| Lactobacillales | 0.48 | -0.34 | 0.57^*^ | -0.71^**^ | 0.57 |
| *Family* |  |  |  |  |  |
| Nitrosotaleaceae | 0.67^*^ | 0.56 | 0.53 | -0.51 | 0.69^*^ |
| Rhodanobacteraceae | 0.03 | -0.10 | 0.17 | 0.01 | 0.17 |
| Sphingomonadaceae | -0.58^*^ | -0.22 | -0.57 | 0.73^**^ | -0.70^*^ |
| Gemmatimonadaceae | -0.10 | -0.29 | -0.10 | -0.06 | -0.04 |
| Pseudomonadaceae | -0.34 | -0.07 | -0.54 | 0.20 | -0.48 |
| Chitinophagaceae | 0.40 | 0.47 | 0.33 | 0.38 | 0.30 |
| SC-I-84 | -0.83^**^ | -0.48 | -0.75^**^ | 0.47 | -0.81^**^ |
| Burkholderiaceae | 0.14 | -0.02 | 0.17 | 0.47 | 0.12 |
| Lactobacillaceae | 0.49 | -0.29 | 0.52 | -0.74^**^ | 0.54 |
| Subgroup_2 | 0.38 | 0.25 | 0.39 | -0.73^**^ | 0.42 |
| *Genus* |  |  |  |  |  |
| *Sphingomonas* | -0.62^*^ | -0.25 | -0.60^*^ | 0.68^*^ | -0.74^**^ |
| *Pseudomonas* | -0.35 | -0.08 | -0.55 | 0.20 | -0.50 |
| *Candidatus_Solibacter* | 0.90^**^ | 0.29 | 0.71^**^ | -0.69^*^ | 0.71^**^ |
| *Bryobacter* | 0.82^**^ | 0.43 | 0.76^**^ | -0.71^**^ | 0.73^**^ |
| *Lactobacillus* | 0.50 | -0.28 | 0.52 | -0.74^**^ | 0.54 |
| *SC-I-84* | -0.83^**^ | -0.48 | -0.75^**^ | 0.47 | -0.81^**^ |

Note: SOC, soil organic carbon; DOC, dissolved organic carbon; N_min_, soil mineral nitrogen; TN, total nitrogen; C/N, soil carbon: nitrogen ratio; MBC, microbial biomass carbon. ^*^indicate significant correlation at *P*<0.05, ^**^ indicate significant correlation at *P*<0.01.

# Table S5 Correlations between fungal alpha-diversity, community composition and enzyme activities

| Correlations | MBC/  g kg^−1^ | β-1,4-xylosidase /  nmol g^−1^ h^−1^ | β-1,4-glucosidase /  nmol g^−1^ h^−1^ | β-D-cellobiohydrolase /  nmol g^−1^ h^−1^ | polyphenol oxidase/  nmol g^−1^ h^−1^ |
| --- | --- | --- | --- | --- | --- |
| Chao1 richness indices | -0.42 | -0.46 | -0.37 | 0.39 | -0.42 |
| Shannon diversity indices | -0.47 | -0.47 | -0.36 | 0.49 | -0.42 |
| *Phylum* |  |  |  |  |  |
| Basidiomycota | -0.65^*^ | 0.29 | -0.57 | 0.58^*^ | -0.61^*^ |
| Ascomycota | 0.22 | -0.27 | 0.38 | -0.03 | -0.13 |
| Mortierellomycota | -0.17 | -0.39 | -0.32 | -0.07 | 0.11 |
| Chytridiomycota | 0.19 | 0.65^*^ | 0.14 | -0.05 | 0.14 |
| Mucoromycota | 0.05 | -0.27 | 0.00 | -0.51 | 0.15 |
| *Class* |  |  |  |  |  |
| Agaricomycetes | -0.72^**^ | 0.23 | -0.57 | 0.62^*^ | -0.65^*^ |
| Sordariomycetes | 0.38 | -0.32 | 0.42 | -0.15 | 0.11 |
| Tremellomycetes | 0.78^**^ | 0.01 | 0.48 | -0.62^*^ | 0.64^*^ |
| Leotiomycetes | -0.29 | 0.13 | -0.39 | 0.15 | -0.15 |
| Mortierellomycetes | -0.17 | -0.39 | -0.32 | -0.07 | 0.11 |
| Rhizophlyctidomycetes | -0.26 | 0.64^*^ | -0.15 | 0.35 | -0.19 |
| Mucoromycetes | 0.08 | -0.27 | 0.01 | -0.53 | 0.19 |
| Dothideomycetes | -0.27 | 0.04 | -0.10 | 0.43 | -0.30 |
| Rhizophydiomycetes | 0.44 | 0.40 | 0.35 | -0.32 | 0.35 |
| Eurotiomycetes | 0.91^**^ | 0.24 | 0.87^**^ | -0.57 | 0.69^*^ |
| *Order* |  |  |  |  |  |
| Phallales | -0.74^**^ | 0.14 | -0.61^*^ | 0.61^*^ | -0.69^*^ |
| Hypocreales | 0.58^*^ | -0.21 | 0.57 | -0.35 | 0.38 |
| Filobasidiales | 0.78^**^ | 0.02 | 0.49 | -0.62^*^ | 0.64^*^ |
| Sordariales | -0.56 | -0.50 | -0.48 | -0.02 | -0.65^*^ |
| Agaricales | 0.36 | -0.12 | 0.54 | -0.27 | 0.32 |
| Mortierellales | -0.17 | -0.39 | -0.32 | -0.07 | 0.11 |
| Mucorales | 0.08 | -0.27 | 0.01 | -0.53 | 0.19 |
| Melanosporales | 0.36 | 0.70^*^ | 0.48 | 0.09 | 0.44 |
| Pleosporales | -0.35 | -0.14 | -0.22 | 0.55 | -0.38 |
| Erysiphales | -0.21 | 0.19 | -0.36 | 0.16 | -0.01 |
| *Family* |  |  |  |  |  |
| Phallaceae | -0.74^**^ | 0.14 | -0.61^*^ | 0.61^*^ | -0.69^*^ |
| Nectriaceae | -0.24 | -0.47 | -0.25 | 0.15 | -0.41 |
| Chaetomiaceae | -0.46 | -0.56 | -0.44 | -0.19 | -0.63^*^ |
| Piskurozymaceae | 0.74^**^ | -0.10 | 0.41 | -0.61^*^ | 0.57 |
| Clavicipitaceae | 0.87^**^ | 0.07 | 0.82^**^ | -0.65^*^ | 0.77^**^ |
| Mortierellaceae | -0.16 | -0.37 | -0.31 | -0.07 | 0.12 |
| Filobasidiaceae | 0.82^**^ | 0.14 | 0.58 | -0.61^*^ | 0.71^**^ |
| Mucoraceae | 0.30 | -0.24 | 0.23 | -0.64^*^ | 0.34 |
| Hypocreaceae | -0.32 | -0.10 | -0.23 | 0.45 | -0.45 |
| *Genus* |  |  |  |  |  |
| *Lysurus* | -0.74^**^ | 0.14 | -0.61^*^ | 0.62^*^ | -0.69^*^ |
| *Fusarium* | -0.33 | -0.53 | -0.33 | 0.17 | -0.54 |
| *Solicoccozyma* | 0.73^**^ | -0.10 | 0.41 | -0.61^*^ | 0.58^*^ |
| *Metacordyceps* | 0.88^**^ | 0.08 | 0.82^**^ | -0.65^*^ | 0.78^**^ |
| *Naganishia* | 0.82^**^ | 0.15 | 0.58^*^ | -0.61^*^ | 0.71^**^ |
| *Mortierella* | -0.19 | -0.37 | -0.33 | -0.11 | 0.11 |

Note: SOC, soil organic carbon; DOC, dissolved organic carbon; N_min_, soil mineral nitrogen; TN, total nitrogen; C/N, soil carbon: nitrogen ratio; MBC, microbial biomass carbon. ^*^indicate significant correlation at *P*<0.05, ^**^ indicate significant correlation at *P*<0.01.

# Table S6 Correlations between functional groups of fungal communities and the abundant classes

| Correlations | Saprotroph | Pathotroph | Pathotroph-Saprotroph | Symbiotroph | Pathogen-Saprotroph-Symbiotroph | Pathotroph-Saprotroph-Symbiotroph | Pathotroph-Symbiotroph | Saprotroph-Symbiotroph |
| --- | --- | --- | --- | --- | --- | --- | --- | --- |
| Agaricomycetes | 0.98^**^ | -0.77^**^ | 0.08 | -0.56 | 0.34 | -0.36 | 0.29 | -0.02 |
| Sordariomycetes | -0.34 | 0.31 | 0.50 | -0.15 | 0.39 | 0.63^*^ | 0.67^*^ | 0.31 |
| Tremellomycetes | -0.88^**^ | 0.76** | -0.41 | 0.64^*^ | -0.56 | 0.11 | -0.52 | -0.17 |
| Leotiomycetes | 0.03 | 0.02 | -0.25 | 0.42 | -0.37 | -0.45 | -0.60^*^ | -0.21 |
| Mortierellomycetes | -0.39 | 0.22 | -0.01 | 0.43 | -0.30 | -0.16 | -0.52 | -0.31 |
| Rhizophlyctidomycetes | 0.62^*^ | -0.41 | -0.28 | -0.27 | 0.12 | -0.30 | -0.01 | 0.27 |
| Mucoromycetes | -0.29 | -0.05 | -0.20 | 0.30 | -0.40 | -0.13 | -0.55 | -0.45 |
| Dothideomycetes | 0.41 | -0.26 | 0.58^*^ | -0.37 | 0.79^**^ | 0.29 | 0.83^**^ | 0.25 |
| Rhizophydiomycetes | -0.10 | 0.16 | -0.37 | 0.12 | -0.28 | -0.29 | -0.06 | -0.40 |
| Eurotiomycetes | -0.77^**^ | 0.80^**^ | -0.28 | 0.52 | -0.26 | 0.24 | -0.06 | 0.38 |

Note: ^*^indicate significant correlation at *P*<0.05, ^**^ indicate significant correlation at *P*<0.01.
